# Cubic C_3_N: A New Superhard Phase of Carbon-Rich Nitride

**DOI:** 10.3390/ma9100840

**Published:** 2016-10-17

**Authors:** Qun Wei, Quan Zhang, Haiyan Yan, Meiguang Zhang

**Affiliations:** 1 School of Physics and Optoelectronic Engineering, Xidian University, Xi’an 710071, China; 2 School of Microelectronics, Xidian University, Xi’an 710071, China; quzhang93@hotmail.com; 3 College of Chemistry and Chemical Engineering, Baoji University of Arts and Sciences, Baoji 721013, China; hyyan1102@163.com; 4 College of Physics and Optoelectronic Technology, Nonlinear Research Institute, Baoji University of Arts and Sciences, Baoji 721016, China

**Keywords:** carbon nitride, first-principles calculations, ideal strength, hardness

## Abstract

Using the particle swarm optimization technique, we proposed a cubic superhard phase of C_3_N (*c*-C_3_N) with an estimated Vicker’s hardness of 65 GPa, which is more energetically favorable than the recently proposed *o*-C_3_N. The *c*-C_3_N is the most stable phase in a pressure range of 6.5–15.4 GPa. Above 15.4 GPa, the most energetic favorable high pressure phase *R*3*m*-C_3_N is uncovered. Phonon dispersion and elastic constant calculations confirm the dynamical and mechanical stability of *c*-C_3_N and *R*3*m*-C_3_N at ambient pressure. The electronic structure calculations indicate that both *c*-C_3_N and *R*3*m*-C_3_N are indirect semiconductor.

## 1. Introduction

Covalent light elements and their compounds are regarded as candidates of superhard materials. In light of the extensive applications of *c*-BN as superhard material, boron nitride compounds, such as *Z*-BN, *Pbca*-BN, *w*-BN, *O*-BN, and *C*222_1_-B_3_N_5_, have been found to be superhard [1,2,3,4,5,6]. Except boron nitrides, carbon nitrides are also expected to be potential superhard materials [7,8,9,10,11,12,13,14,15]. Since the prediction of hexagonal *β*-C_3_N_4_ with extraordinary hardness, extensive experimental, and theoretical investigations have been carried out to search for new C–N materials with novel properties. A following work [9] proposed that the hardness of *c*-C_3_N_4_ is close to that of diamond using the microscopic model of hardness. In recent years, several different crystal structure prediction technique-based algorithms have been the most prevalent strategies to explore and predict new material structures. Using the swarm optimization technique, Li et al. [13] found a novel low compressible and superhard *bct*-CN_2_ phase. Zhang et al. predicted that the stable CN is *P*4_2_/*m* phase [11]. A phase diagram of carbon nitrides was predicted by Dong et al. [12] in the pressure range of 0–300 GPa. Replacing atoms is another common way to find new phases. By substituting all B sites and three N sites out of five in *C*222_1_-B_3_N_5_ by C atoms, Hao et al. proposed a *C*222_1_ superhard phase *o*-C_3_N with a Vicker’s hardness of 76 GPa [16], and this *o*-C_3_N is more energetically favorable than the previously proposed C_3_N phases [17,18]. Although different kinds of structures have been proposed, the quest for a new energetically stable or metastable C_3_N phase is a hot topic in superhard materials. In the present work, we performed an extensive structure search for the most energetically stable high pressure C_3_N phases using the prevalent developed crystal structure analysis by particle swarm optimization (CALYPSO) package [19]. This method has been successfully applied to predict structures that have been confirmed by experiments [8,20,21,22]. Two high-pressure phases with group symmetry *Fd*-3*m* and *R*3*m* phases were uncovered, and the pressure-induced phase transition was then found. The crystal structures, electronic structures, and mechanical behaviors of these two phases were systematically studied.

## 2. Results and Discussion

**Figure 1:**
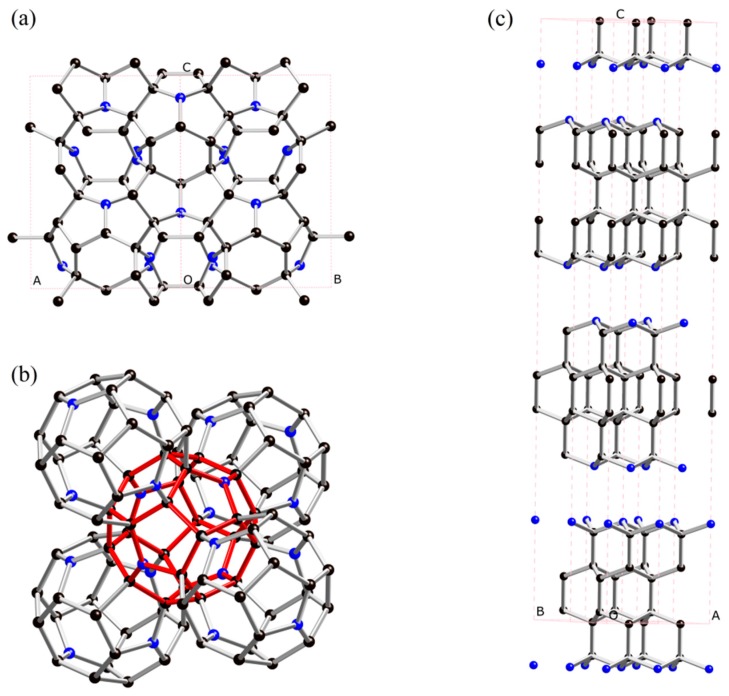
Crystal structure of *c*-C_3_N in [100] view (**a**); cages in *c*-C_3_N (**b**); and 2 × 1 × 1 supercell of *R*3*m*-C_3_N (**c**). The blue and black balls represent the N and C atoms, respectively. To clearly show the relative position relation of cages, the central cage is shown in red.

At ambient pressure, the ground state of C_3_N revealed by our structural search is confirmed to be the *P*6/*mmm* phase (*h*-C_3_N) proposed in [16]. For high pressures at 10 GPa and 20–100 GPa, our structure searches found the most stable structures to be a cubic *Fd*-3*m* phase (hereafter denoted as *c*-C_3_N, see Figure 1a) and a layered hexagonal *R*3*m* phase (see Figure 1c), respectively. Meanwhile, the recent proposed orthorhombic *C*222_1_ phase (*o*-C_3_N) was also explored as a metastable phase. As shown in Figure 1a, the *c*-C_3_N contains 128 atoms in its unit cell, in which the C and N atoms occupying the 96g and 32e positions. In more detail, for *c*-C_3_N, each N atom is sp^2^-hybridized and connects with three C atoms with a C–N bond length of 1.429 Å. Meanwhile, each C atom is connected with three C atoms and one N atom with two 1.551 Å C–C bonds, one 1.623 Å C–C bond, and one 1.429 Å C–N bond. Interestingly, as seen in Figure 1b, the *c*-C_3_N consists of a fundamental building block: a C24N4 cage is composed of four C6 regular hexagon and twelve C4N pentagons. These cages are connected by sharing a C6 hexagon. The crystal structure of *R*3*m*-C_3_N is shown in Figure 1c. There are 24 atoms in one unit cell, including three layers. Each layer contains two N atoms and six C atoms. N atoms are bonded with three C atoms, and the C–N bond length is 1.503 Å. Detailed structural information of the predicted *c*-C_3_N and *R*3*m*-C_3_N at ambient pressure is presented in Table 1.

**Figure 2:**
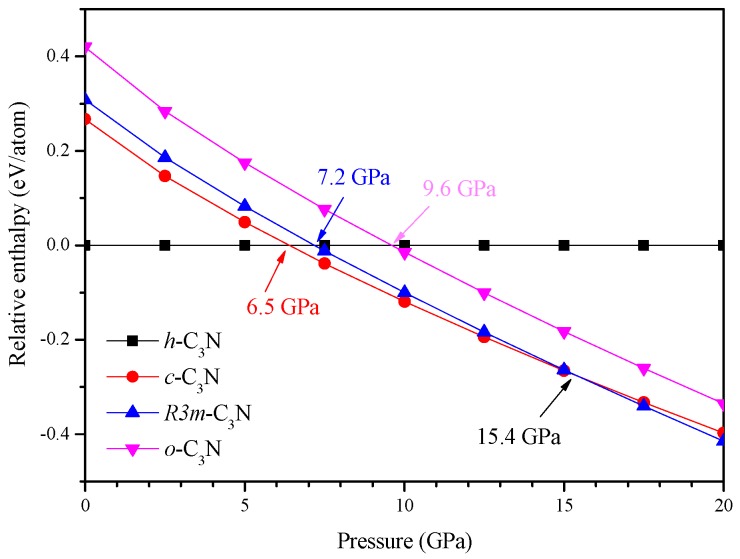
Enthalpies differences of *c*-C_3_N, *R*3*m*-C_3_N, and *o*-C_3_N relative to *h*-C_3_N as a function of pressure.

**Figure 3:**
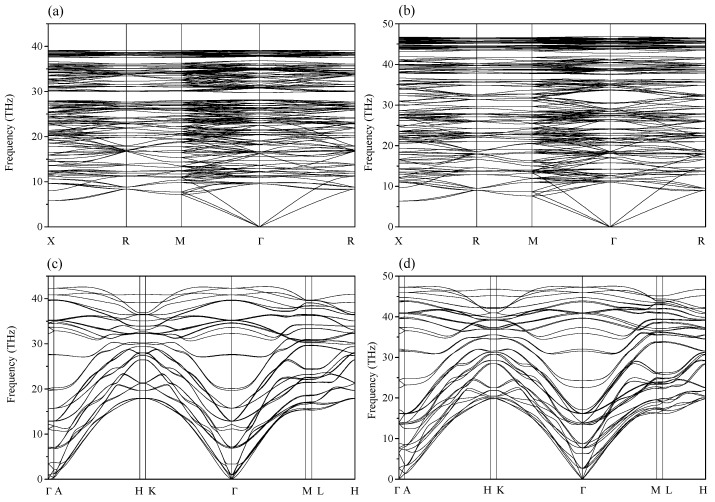
Phonon dispersion of *c*-C_3_N at 0 GPa (**a**) and 100 GPa (**b**) and *R*3*m*-C_3_N at 0 GPa (**c**) and 100 GPa (**d**).

To determine the phase transition pressure of C_3_N, the enthalpies differences of the predicted *c*-C_3_N, *R*3*m*-C_3_N, and *o*-C_3_N relative to *h*-C_3_N as a function of pressure are illustrated in Figure 2 up to 20 GPa. It should be noted that, in the 20–100 GPa pressure range, the enthalpies of these phases follow the same order as that at 20 GPa. To clearly show the phase transition pressure, in Figure 2, the pressure range is taken as 0–20 GPa. It can be seen from Figure 2 that the predicted *c*-C_3_N becomes more stable than *h*-C_3_N above 6.5 GPa, whereas the transition pressures from *h*-C_3_N to *R3m*-C_3_N and *o*-C_3_N are 7.2 and 9.6 GPa, respectively. In the 6.5–15.4 GPa pressure range, *c*-C_3_N is the most stable phase. Above 15.4 GPa, it transforms to the *R*3*m* phase. As seen, the previous proposed *o*-C_3_N is a metastable phase in the whole considered pressure range. To verify the stability of the newly proposed phases, we have performed the phonon calculations of *c*-C_3_N and *R*3*m*-C_3_N at 0 and 100 GPa, and the results are illustrated in Figure 3. As shown in Figure 3, the absence of imaginary phonon frequency in the whole Brillouin zone indicates their dynamical stability up to at least 100 GPa.

To study the mechanical properties of *c*-C_3_N, the elastic constants were calculated using the strain–stress method. The average bulk modulus, shear modulus, and Young’s modulus of *c*-C_3_N were further estimated using the Voigt–Reuss–Hill approximation. The calculated results are listed in Table 2, together with the elastic parameters of *o*-C_3_N and diamond for comparison. Although the bulk modulus of *c*-C_3_N (344 GPa) is less than that of *o*-C_3_N (404 GPa) and diamond (433 GPa), it is comparable to that of *P*4_2_/*m*-CN (341 GPa), *Pnnm*-CN (369 GPa), and BeC_2_N (315 GPa for CH and 311 GPa for WU structure) [11,23], indicating the ultra-incompressibility of *c*-C_3_N. The elastic constants of *c*-C_3_N satisfy the criteria for a cubic crystal [C_11_ − C_12_ > 0, C_11_ > 0, C_44_ > 0], showing that it is mechanically stable at ambient pressure. By using Chen’s model [24], the Vicker’s hardness is 64.6 GPa estimated by $H_{V}=2{\left(G^{3}/B^{2}\right)}^{0.585}-3$, which means *c*-C_3_N is a potential superhard material.

**Figure 4:**
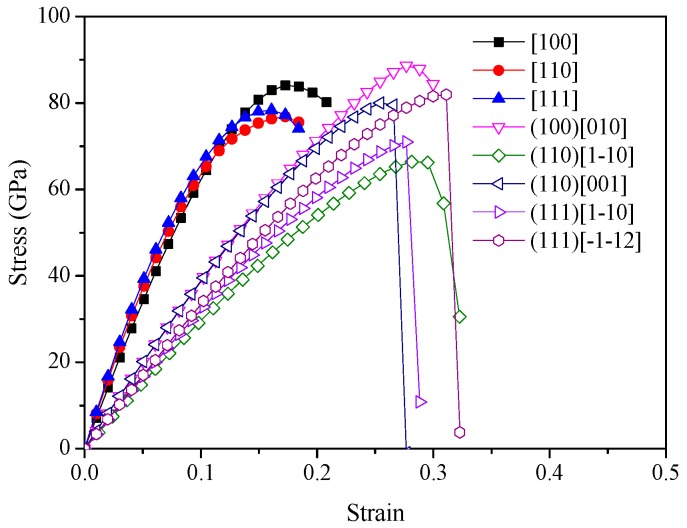
Strain–stress relations for *c*-C_3_N in various tensile and shear directions.

The ideal strength in a specified direction is microscopically determined by bond strength and breaking nature under strain. Thus, the comprehensive analysis of the bonding nature and of the ideal strength can provide a deeper insight into mechanical behavior and the hardness of materials. The tensile strain–stress relations can be obtained through the projection of the unitcell onto the corresponding crystal axes with one axis parallel to the stain direction for tension deformation. Figure 4 shows the ideal tensile and shear strength of *c*-C_3_N. The ideal tensile strength for *c*-C_3_N are calculated in the main high symmetry directions, including [100], [110], and [111]. The lowest ideal tensile strength appears in the [110] direction with 76.8 GPa. This means, under tensile loadings, *c*-C_3_N would first cleave in the (110) plane. The ideal shear strength was then calculated by applying the [1–10] and [001] shear deformations in the (110) easy cleavage plane perpendicular to the weakest tensile direction. The ideal shear deformations in the (100)[010], (111)[1–10], and (111)[–1–12] are also shown in Figure 4 for comparison. The lowest peak shear stress occurs in the (110)[1–10] direction with 66.4 GPa, which is lower than the lowest tensile strength. This means the failure mode in *c*-C_3_N is dominated by the shear type with 66.4 GPa. This value is also close to the hardness calculated by Chen’s model.

**Figure 5:**
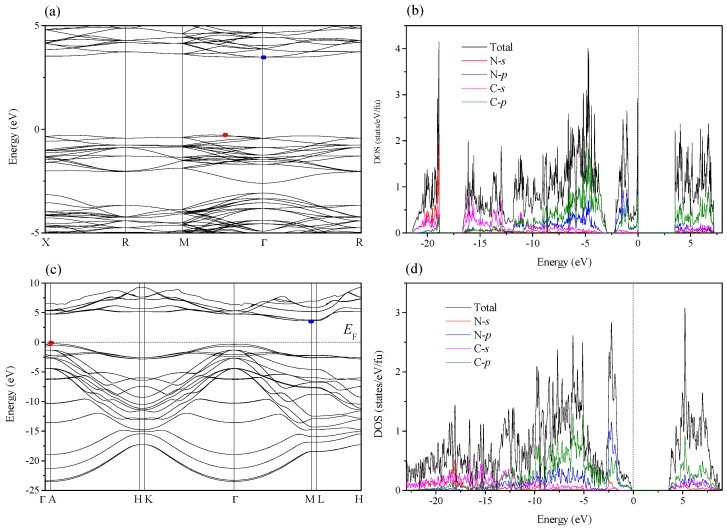
Band structure of *c*-C_3_N (**a**); DOS of *c*-C_3_N (**b**); band structure of *R*3*m*-C_3_N (**c**); and DOS of *R*3*m*-C_3_N (**d**); the red points represent the valence band maximum (VBM) and the blue points represent the conduction band minimum (CBM); the dotted lines represent the Fermi level. DOS: density of states.

Figure 5 shows the band structure and density of states (DOS) of *c*-C_3_N and *R*3*m*-C_3_N. As seen in Figure 5a, *c*-C_3_N is an indirect band gap semiconductor with 3.76 eV gap. The conduction band minimum (CBM) is located at Г point, whereas the valence band maximum (VBM) is located at (0.2 0.2 0.0) along the M-Г direction. As indicated by the partial DOS in Figure 5b, the peaks at low-energy range (below −12 eV) are mainly attributed to C-2s and N-2s states. In the energy range of −12 to 0 eV, the contribution from C-2p and N-2p states are much larger than that of C-2s and N-2s states. Near the Fermi level, the contributions from C-2p and N-2p are almost the same, which indicates the significant hybridization between these two orbitals. For *R*3*m*-C_3_N, the CBM is located at M point and the VBM is at A point, which leads to an indirect band gap semiconductor with 3.85 eV gap, as seen in Figure 5c. Similar to *c*-C_3_N, there is a significant hybridization between C-2p and N-2p orbitals due to the nearly equal values near the Fermi level (see Figure 5d).

## 3. Computational Methods

The crystal structure prediction is based on the global minimization of energy surfaces merging ab initio total energy calculations as implemented in CALYPSO code [19]. We performed variable cell structure predictions at 0, 10, 20, 50, and 100 GPa with one to eight formula units (f.u.) per simulation cell. At each pressure point, we found 30 generations and 40 structures generated via particle swarm optimization (PSO) per generation. For structural relaxations and electronic calculations, various first-principles calculation methods have been used in carbon-based systems [25,26,27]. In the present work, we performed structural relaxations and electronic calculations within the density functional theory, carried out within the Vienna ab initio simulation package (VASP) [28], with the projector augmented wave method [29]. The electronic wave functions were expanded in a plane-wave basis set with a well converged cutoff energy of 900 eV. Monkhorst–Pack *k* point meshes with a grid of 0.02 A^−1^ for Brillouin zone sampling were chosen to ensure that total energies converged were better than 1 meV/atom. The phonon frequencies were calculated by a supercell approach as implemented in PHONOPY code [30], and the forces were calculated from VASP. The elastic constants were calculated by the strain–stress method, and Young’s modulus, the shear modulus, the bulk modulus, and Poisson’s ratio were derived from the Voigt–Reuss–Hill approximation [31].

## 4. Conclusions

In summary, by using unbiased structure searching techniques in combination with first-principles calculations, we have predicted two new high pressure phases of C_3_N: *c*-C_3_N and *R*3*m*-C_3_N. Both *c*-C_3_N and *R*3*m*-C_3_N are dynamically stable in the pressure range from 0 to 100 GPa. The pressure induced a phase transition in the following order: *h*-C_3_N → *c*-C_3_N → *R*3*m*-C_3_N. The transition pressure was 6.5 and 15.4 GPa, respectively. The high bulk modulus of *c*-C_3_N shows that *c*-C_3_N is ultra-incompressible. The Vicker’s hardness of *c*-C_3_N was estimated to be about 65 GPa. The ideal strength calculations indicate that *c*-C_3_N is intrinsically superhard. The failure mode in *c*-C_3_N was dominated by the shear type with 66.4 GPa. Both *c*-C_3_N and *R*3*m*-C_3_N are indirect band gap semiconductors at ambient pressure with 3.76 and 3.85 eV gaps, respectively. Such theoretical predictions can guide future synthesis experiments of carbon-rich nitrides.

## Figures and Tables

**Table 1:** The calculated equilibrium structural parameters of *c*-C_3_N and *R*3*m*-C_3_N at ambient pressure.

Phase	*a*, *b*, *c* (Å)	Atomic Coordinates	*d*_C–C_ (Å)	*d*_C–N_ (Å)
*c*-C_3_N	*a* = 9.5342	C 96g (0.93983, 0.93983, 0.75924) N 32e (0.35488, 0.35488, 0.35488)	1.551 1.623	1.429
*R*3*m*-C_3_N	*a* = 2.4519 *c* = 31.7508	C 3a (0.33333, 0.66667, 0.07030) C 3a (0.33333, 0.66667, 0.02080) C 3a (0.33333, 0.66667, 0.28862) C 3a (0.66667, 0.33333, 0.08675) C 3a (0.66667, 0.33333, 0.00436) C 3a (0.66667, 0.33333, 0.13582) N 3a (0.66667, 0.33333, 0.27269) N 3a (0.00000, 0.00000, 0.15174)	1.558 1.572 1.553 1.509	1.503

**Table 2:** The calculated elastic constants *C*_ij_ (GPa), Young’s modulus *E* (GPa), bulk modulus *B* (GPa), shear modulus *G* (GPa), and the *G*/*B* ratios of *o*-C_3_N, *c*-C_3_N, and diamond.

Crystal	Reference	*C*_11_	*C*_22_	*C*_33_	*C*_44_	*C*_55_	*C*_66_	*C*_12_	*C*_13_	*C*_23_	*E*	*B*	*G*	*G*/*B*
*o*-C_3_N	This work	1105	806	804	484	424	424	122	125	222	-	404	409	1.01
-	[16]	1092	797	796	482	398	392	131	132	222	918	406	401	0.99
*c*-C_3_N	This work	766	-	-	403	-	-	133	-	-	810	344	365	1.06
Diamond	[4]	1052	-	-	563	-	-	120	-	-	-	431	522	1.2
